# Characterization and performance of the Allegro™ STR 200 single-use stirred tank bioreactor

**DOI:** 10.1186/1753-6561-9-S9-P61

**Published:** 2015-12-14

**Authors:** Byron Rees, John Thompson, Bojan Isailovic, Kerem Irfan, Camille Segarra

**Affiliations:** 1 Pall Corporation, 5 Harbourgate Business Park, Southampton Road, Portsmouth, PO6 4BQ, UK

## Background

Currently, stirred tank reactors (STR) represent the gold standard for the large scale growth of suspension cell lines. Culture performance is strongly influenced by the efficiency of mixing, as measured by the mass transfer coefficient and mixing time. The success of traditional stainless steel or glass STR systems lies in their impeller driven agitation that efficiently mixes large volumes of culture fluid. However, the transition from stainless and glass STR vessels to single use STR systems still remains a challenge, particularly in terms of replicating efficient mixing. Many single use STRs use magnetically coupled driving mechanism to eliminate the risks of biocontainer leaks from rotating mechanical seals. While this design mitigates the risks of compromising sterility, insufficient magnet strength limits the power that can be transmitted to the culture fluid, and often results in poor mixing and mass transfer performance.

## Materials and methods

All experiments involving measurement were carried out using probes as follows:

• pH, DO and conductivity were measured using two probes, located at two different locations.

• Probe 1 was located at the bottom of the biocontainer, through the standard probe port.

• Probe 2 was located at the back left corner of the biocontainer. This location was identified as a "worst area" by doing a decolorization method using a pH indicator dye.

• Temperature was measured using 10 thermocouples, at various locations within the biocontainer.

## Results

**Figure 1:**
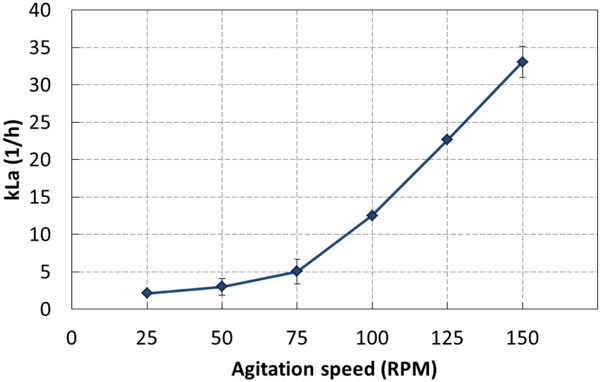
**kLa 2080 values at various agitation rates**.

The Allegro STR 200 single-use stirred tank bioreactor is characterized by excellent mixing performance thanks to a direct drive agitation mechanism coupled with a large bottom-mounted impeller, a square shaped biocontainer with natural baffling effects, as well as integrated baffles. The relationship between Kla and agitation speed for the unit are shown in figure 1.

In terms of temperature studies, fluid was heated from 4 ºC to 37 ºC in 8.88 hours and was shown to be stable at 37 ºC ± 0.1 ºC at 200 L scale.

## Conclusions

The unique features of the Allegro STR 200 single-use stirred tank bioreactor allow for: a wide range of power input per unit volume of fluid, mixing times below 10 seconds, oxygen transfer rate (kLa) as high as 33h^-1^using a ring sparger and air, efficient CO_2 _stripping, precise and homogenous temperature control.

